# Metabolic acidosis in patients with kidney disease

**DOI:** 10.1590/2175-8239-JBN-2020-0139

**Published:** 2020-08-10

**Authors:** Silvia J Leon, Navdeep Tangri

**Affiliations:** 1 Seven Oaks General Hospital, Chronic Disease Innovation Centre, Winnipeg, Manitoba, Canada.; 2 University of Manitoba, Max Rady College of Medicine, Department of Community Health Sciences, Winnipeg, Manitoba, Canada.; 3 University of Manitoba, Department of Internal Medicine, Max Rady College of Medicine, Winnipeg, Manitoba, Canada.

The number of people receiving renal replacement therapy (RRT), through either dialysis or renal transplantation, exceeds 2.5 million worldwide and its prevalence is expected to rise sharply over the next decades^1^. The predicted growth in prevalence of end-stage kidney disease (ESKD) demonstrates a need for initiatives that will slow the progression to ESKD in Chronic Kidney Disease (CKD) and preserve quality and quantity of life from those on dialysis.

For more than a century, metabolic acidosis has been recognized as a complication of CKD. In the last decade, studies have shown that metabolic acidosis in an independent risk factor for CKD progression, bone loss, and muscle metabolismo^2^^,^ 3 ^,^ 4 and studies evaluating the benefits of treating metabolic acidosis have increased steadily.

Metabolic acidosis is defined as serum bicarbonate levels that are persistently less than 22 mEq/L^5^. The prevalence and severity of metabolic acidosis increases as kidney disease progresses, and metabolic acidosis is associated with several adverse outcomes that varies into severity. Among the described adverse outcomes are worsening of bone and muscle health, hyperkalemia, insulin resistance, progression of CKD, and an increased risk of mortality^5^.

Clinical guidelines for the management of CKD recommend the treatment of metabolic acidosis (serum bicarbonate <22 mEq/L) with oral alkali^6^. However, treatment of metabolic acidosis with oral alkali in patients with CKD is lower than expected. In the CRIC study, only 2.7% of patients stage 2-4 CKD with serum bicarbonate <22 mmol/L were receiving treatment with an oral alkali^4^.

In patients receiving hemodialysis, studies have shown that metabolic acidosis is the most common acid-base abnormality found in this population. To date, there is no consensus whether the target serum bicarbonate levels should vary by dialysis modality. K/DOQI guidelines recommend serum bicarbonate levels ≥22mEq/L irrespective of the dialysis modality^6^.

Despite the risk associated with metabolic acidosis and the benefits of treating it, a large proportion of patients receiving hemodialysis have suboptimal correction of metabolic acidosis, and maintenance dialysis therapies are often not able to completely correct the base deficit. It has been suggested that correction of metabolic acidosis results in a decrease in hospitalizations and mortality in patients receiving dialysis. To understand the possible impact of drug therapies for metabolic acidosis, it is important to know the burden of metabolic acidosis in patients with impaired renal function.

The report by Silva *et al.* 7 in this issue of the *Brazilian Journal of Nephrology* addresses this important topic and adds an additional reason to consider treating metabolic acidosis. Using a cross-sectional study, these investigators examined the prevalence of metabolic acidosis in patients receiving hemodialysis in Rio de Janeiro, Brazil. Three hundred and eighty-four patients were included, and blood gas analyses were performed before a midweek dialysis session. Samples were collected from the arteriovenous fistula or directly from the central intravenous catheter. The mean total CO_2_ was 22.7 ±3.0 mEq/L, and 40.3% had values lower than 22 mEq/L. Participants received similar concentration of bicarbonate in the dialysis fluid (31.4 mEq/L in all but one center who used 32.4 mEq/L). It is worth to consider that the study by Silva *et al.* does not mention measures of acid-base status beyond total CO_2_ that has been highlighted as a limitation of other research, as we cannot evaluate acid-base disturbances from an isolated total CO_2_ measurement nor can exclude patients with respiratory alkalosis for example^8^. Moreover, total CO_2_ was measured only once per patient which could lead to bias due to measurement error.

In multivariate logistic regression models, older age and standard Kt/V were significantly associated with lower risk of total CO_2_ <22 mEq/L. As the authors acknowledge, there are no previous reports evaluating the association of Kt/V and bicarbonate levels^7^. Future investigations addressing this association will be informative.

As mentioned earlier, treatment of metabolic acidosis remains low in patients with CKD. In patients with CKD, treatment of metabolic acidosis is based on 2 major treatment strategies: 1) dietary recommendations, 2) oral sodium-based alkali. In the last year, a new non-absorbed, counterion-free, polymeric drug that selectively binds and removes hydrochloric acid from the gastrointestinal lumen, has shown to be safe and effective for the treatment of metabolic acidosis in patients with CKD in clinical trials^9^. In patients on hemodialysis, a higher serum bicarbonate in the dialysate fluid can be used to treat acidosis, but it is important to prevent alkalosis and therefore careful monitoring of acid-base status is required.

There are several potential benefits to treatment of acidosis in the hemodialysis population that could be realized. Although these patients have already progressed to dialysis, correction of acidosis may improve their bone health and physical and cognitive functions. Studies from the CKD population show that muscle function can improve with only 3 months of treatment, and physical function-related quality of life also shows a sustained improvement. Acidosis has also been linked to cognition, and given the cognitive deficits seen in the dialysis population, treatment may be beneficial and deserves consideration. Finally, patients on hemodialysis have an increased risk of fractures, and preserving acid base balance may prevent bone loss and subsequent fracture events.

In conclusion, chronic metabolic acidosis is frequently found in patients with CKD and ESKD and is often undertreated. The study by Silva *et al.* is an interesting study that will hopefully bring the attention to the importance of the measurement of serum bicarbonate levels and its appropriate treatment in the care of patients with CKD and kidney failure requiring hemodialysis.
